# Sesquiterpene Alcohol Cedrol Chemosensitizes Human Cancer Cells and Suppresses Cell Proliferation by Destabilizing Plasma Membrane Lipid Rafts

**DOI:** 10.3389/fcell.2020.571676

**Published:** 2021-01-21

**Authors:** Siddhartha Kumar Mishra, Yun Soo Bae, Yong-Moon Lee, Jae-Sung Kim, Seung Hyun Oh, Hwan Mook Kim

**Affiliations:** ^1^Gachon Institute of Pharmaceutical Sciences, Gachon University, Incheon, South Korea; ^2^Cancer Biology Laboratory, Department of Zoology, School of Biological Sciences, Dr. Harisingh Gour Central University, Sagar, India; ^3^Department of Life Sciences, Chhatrapati Shahu Ji Maharaj University, Kanpur, India; ^4^Department of Life Science, College of Natural Sciences, Ewha Womans University, Seoul, South Korea; ^5^Department of Manufacturing Pharmacy, College of Pharmacy, Chungbuk National University, Cheongju, South Korea; ^6^Department of Surgery, University of Florida, Gainesville, FL, United States

**Keywords:** cholesterol, sphingolipid, membrane transport proteins, drug combination, β-cyclodextrins, cedrol (PubChem CID: 65575)

## Abstract

Chemosensitization of cancer cells with small molecules may improve the therapeutic index of antitumoral agents by making tumor cells sensitive to the drug regimen and thus overcome the treatment resistance and side effects of single therapy. Cell membrane lipid rafts are known to transduce various signaling events in cell proliferation. Sensitizing cancer cells may cause modulation of membrane lipid rafts which may potentially be used in improving anticancer drug response. Cedrol, a natural sesquiterpene alcohol, was used to treat human leukemia K562 and colon cancer HT-29 cell lines, and effects were observed. Cedrol decreased the cell viability by inducing apoptosis in both cell lines by activation of pro-apoptosis protein BID and inhibition of anti-apoptosis proteins Bcl-X_*L*_, Bcl-2, and XIAP. Cedrol activated the caspase-9-dependent mitochondrial intrinsic pathway of apoptosis. Furthermore, cedrol inhibited the levels of pAKT, pERK, and pmTOR proteins as well as nuclear and cytoplasmic levels of the p65 subunit of NF-κB. Cedrol caused redistribution of cholesterol and sphingomyelin contents from membrane lipid raft, which was confirmed by a combined additive effect with methyl-β-cyclodextrin (lipid raft-disrupting agent). Lipid raft destabilization by cedrol led to the increased production of ceramides and inhibition of membrane-bound NADPH oxidase 2 enzyme activity. Cholesterol/sphingomyelin-redistributing abilities of cedrol appear as a novel mechanism of growth inhibition of cancer cells. Cedrol can be classified as a natural lipid raft-disrupting agent with possibilities to be used in general studies involving membrane lipid raft modifications.

## Introduction

Cancer is the result of a stepwise progressive disruption of cellular signaling cascades controlling cell proliferation, survival, and differentiation (Evan and Vousden, 2001). The target of most of the currently known anticancer drugs is to reactivate apoptotic cascade and render the cell apoptosis prone so that low doses of cytotoxic drugs can also cause apoptosis (Blagosklonny, 2004). Identification of novel cellular sensitization methods exerting multiple effects on cancer cells growth has become an important aspect of anticancer therapeutic strategies. Certain signals related to cell growth and other physiological processes are transduced through discrete regions in the plasma membrane known as lipid rafts (Edidin, 2001; van Deurs et al., 2003). Cancer cell sensitization by chemotherapeutic candidates mainly targets apoptosis reactivation through intervening a variety of cellular signaling pathways (Shabbits et al., 2003). Some of the commonly targeted cellular mechanisms include increased DNA repair activity (Kelley et al., 2014), regulating cell cycle checkpoints (Visconti et al., 2016), regulating redox balance (Helfinger and Schröder, 2018), modifying p53 activity (Ferreira et al., 1999), regulating the expression of the Bcl2 family (Hata et al., 2015), regulating drug efflux pumps, and altering drug target enzymes (Shabbits et al., 2003; Ughachukwu and Unekwe, 2012). This also includes the breakdown of sphingolipid product ceramide as considerable interest toward apoptosis and coordinated cellular stress responses (Huang et al., 2011). Lipid raft microdomains are characterized by light buoyant density and relatively high levels of glycosphingolipids and cholesterol (van Deurs et al., 2003). Alterations in cholesterol–lipid raft have been directly associated with the activation of multiple pathways including but not limited to protein kinase C (PKC) activation or deactivation and apoptotic membrane receptor activation, and through an increase in intracellular calcium levels (George and Wu, 2012). Lipid rafts mediate the pro-survival signaling, mainly phosphatidylinositol 3’-kinase (PI3K)/AKT pathways that regulate the phosphorylation of the pro-apoptotic proteins and upregulate the expression of the anti-apoptotic genes such as Bcl-X_*L*_ and FLICE-inhibitory protein (Malorni et al., 2008; George and Wu, 2012). Lipid rafts contain ceramide, a sphingolipid messenger, which initiates apoptotic functions upon activation and regulates the tumor cell fate (Megha and London, 2004). High metabolic activity in cancer cells causes overproduction of reactive oxygen species (ROS) which further activates a variety of redox-sensitive transcription factors including nuclear factor kappa-light-chain-enhancer of activated B cells (NF-κB) (Gajate et al., 2009). Upon activation, it augments the pro-survival functions by activating ERK/MEK and the PI3K/AKT pathways. ROS generation in cancer cells is also observed through a non-mitochondrial source involving membrane-bound NADPH oxidases (Leto et al., 2009; Bae et al., 2011). Nox2 and Nox4, located in lipid raft regions of the cell membrane, have been associated with growth promotion in cancer cells (Leto et al., 2009; Jeon et al., 2010; Rao Malla et al., 2010). Molecular linkages between cell death, cell survival, and cell cycle have become an object of intense research in recent years. The intrigued role of lipid rafts in transducing cell survival and death processes indicates that alterations in the integrity of the membrane lipid rafts may directly interfere with the cell growth.

Natural products have currently been considered as primary sources for chemotherapeutic agents with promising antitumor activities. Sesquiterpenes are a group of natural terpenoids exhibiting a wide range of bioactivities including anti-inflammatory, cytotoxic, and anti-proliferative effects (Zhang et al., 2005). As the tumors are known to develop resistance to chemotherapeutic drugs, the effective chemosensitizers are explored from different sources including dietary and natural agents. Terpenoids like resveratrol, artemisinin, thapsigargin, parthenolide, and andrographolide have shown promising pharmacological abilities to inhibit the growth of cancer cells through modulation of several key signaling pathways especially cell cycle, apoptosis, growth and proliferation, cell adhesion, migration and invasion, and metastasis (Zhang et al., 2005; Reis-Sobreiro et al., 2009; Ghantous et al., 2010; Mishra et al., 2015; Islam et al., 2018). Some phenolic derivatives and flavonoids like curcumin, resveratrol, and epigallocatechin-3-gallate have shown potent combinatorial treatment efficacy in preclinical and clinical chemotherapeutic settings (Gezici and Şekeroğlu, 2019). They have shown lower toxicity and immediate availability, which led to strong antitumor activity through modulation of several key signaling pathways mainly cell proliferation, invasion, metastasis, and angiogenesis (Ghantous et al., 2010; Gupta et al., 2011). Resveratrol has been utilized for chemosensitization in different cancers such as lung cancer, prostate cancer, multiple myeloma, acute myeloid leukemia, promyelocytic leukemia, oral carcinoma, and pancreatic adenoma (Reis-Sobreiro et al., 2009; Gupta et al., 2011). Chemotherapeutic agents like 5-fluorouracil (5-FU), doxorubicin, paclitaxel, cisplatin, gefitinib, gemcitabine, and vincristine have shown enhanced effects when treated in combination with resveratrol. This chemosensitization effect was mediated largely through modulation of multiple-cell signaling pathways including drug transporters, cell proliferation and survival, and specific signaling pathways via NF-κB and STAT-3 (Gupta et al., 2011). Cedrol is a natural crystalline sesquiterpene alcohol found in the essential oil of conifers especially cedar wood (*Juniperus virginiana*). The antioxidant activity of the essential oil of propolis containing cedrol was detected recently (Chi et al., 2020). The pharmacokinetics and pharmacodynamics of cedrol polymorphs were associated with anti-inflammatory and analgesic effects in a mouse paw edema model induced by xylene (Wang et al., 2019). Cedrol when treated to LPS-induced inflammation in C57BL/6J mice showed that pro-inflammatory cytokines (TNF-α or IL-1β) were reduced and the compensatory anti-inflammatory cytokine IL-10 was activated (Millet et al., 2018). Cedrol inhibited proliferation of lung cancer A549 cells through the mitochondrial transmembrane protein and suppression of PI3K/Akt signaling pathways (Zhang et al., 2016). We aimed to investigate the chemosensitizing and growth inhibitory abilities of cedrol on proliferation of human leukemia and colon cancer cells and to elucidate the molecular mechanism underlying the effects of cedrol. A remarkable observation to note is that cedrol caused redistribution of cholesterol and sphingomyelin from membrane lipid rafts and led to growth inhibition of cancer cells.

## Results

### Cedrol Induced Apoptotic Cell Death in Human Cancer Cells

**FIGURE 1:**
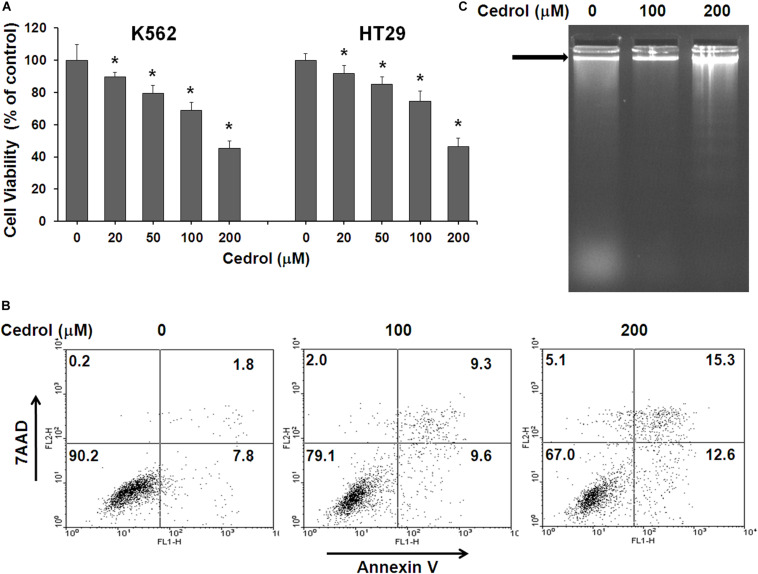
Effect of cedrol on growth of K562 and HT29 cells. **(A)** K562 and HT29 cells were treated with vehicle control (0.1% DMSO) and indicated concentrations of cedrol for 48 h. Cell viability was assayed as described in Materials and Methods, and viable cells were denoted as a percentage of vehicle controls (VH). The data are expressed as means ± SD of six wells from the 96-well plate, and the whole experiment was independently repeated for three times. *P vs. VH. **(B)** K562 cells were treated with VH or cedrol for 18 h and then stained with annexin-V/7AAD. Apoptosis was detected for flow cytometric analysis. *P vs. VH; #*P* vs. VH + z-VAD-fmk. **(C)** K562 cells were treated with cedrol for 18 h and then analyzed for DNA fragmentation by agarose gel electrophoresis.

In order to investigate the effect of cedrol on growth of human cancer cells, we treated a panel of human cancer cell lines and assayed the cell viability. Cedrol suppressed the growth of each cell line in a dose-dependent manner (Supplementary Table S1). Human leukemia cell K562 and colon cancer cell HT29 were found to be most sensitive among them with growth inhibitory (GI50) concentrations 179.5 and 185.5 μM, respectively. Cedrol drastically suppressed the growth of K562 and HT29 cells at 100 and 200 μM concentrations (Figure 1A). Next, we examined the apoptosis-inducing activity of cedrol in K562 cells by annexin V/7-7-amino-actinomycin (AAD) double staining and analyzing DNA contents by flow cytometry. Annexin V is a calcium-dependent phospholipid-binding protein with high-affinity phosphatidylserine, which makes it useful for analyzing apoptotic cells that are exposed with phosphatidylserine, while 7-AAD is a standard probe for cell viability that distinguishes between viable from non-viable cells, thus preferably used in combination with annexin V. Cedrol treatment caused high annexin V staining of cells, considered as apoptotic cells (Figure 1B). Results also show that cedrol partially induced necrosis in K562 cells by 2% (100 μM) and 5.1% (200 μM) (Figure 1B). The pro-apoptotic activity of cedrol in K562 cells was also evident from an early apoptosis marker, DNA strand fragmentation. The band pattern on agarose gel indicates the remarkable increase in DNA fragmentation (∼200 bp) especially at 200 μM cedrol (Figure 1C). The effect of cedrol on cell cycle progression in K562 cells was also observed (Supplementary Figure S1). Propidium iodide (PI) staining of K562 cells showed increased accumulation of cells in the sub-G1 phase with increasing concentrations of cedrol by 5% (100 μM) and 18.4% (200 μM). Although this result supports the pro-apoptosis effect of cedrol, the effect on cell cycle progression was not clear. Collectively, these results demonstrate that cedrol suppresses growth of human cancer cells by inducing apoptosis.

### Cedrol Induced Mitochondrial Intrinsic Apoptosis in K562 and HT29 Cells

**FIGURE 2:**
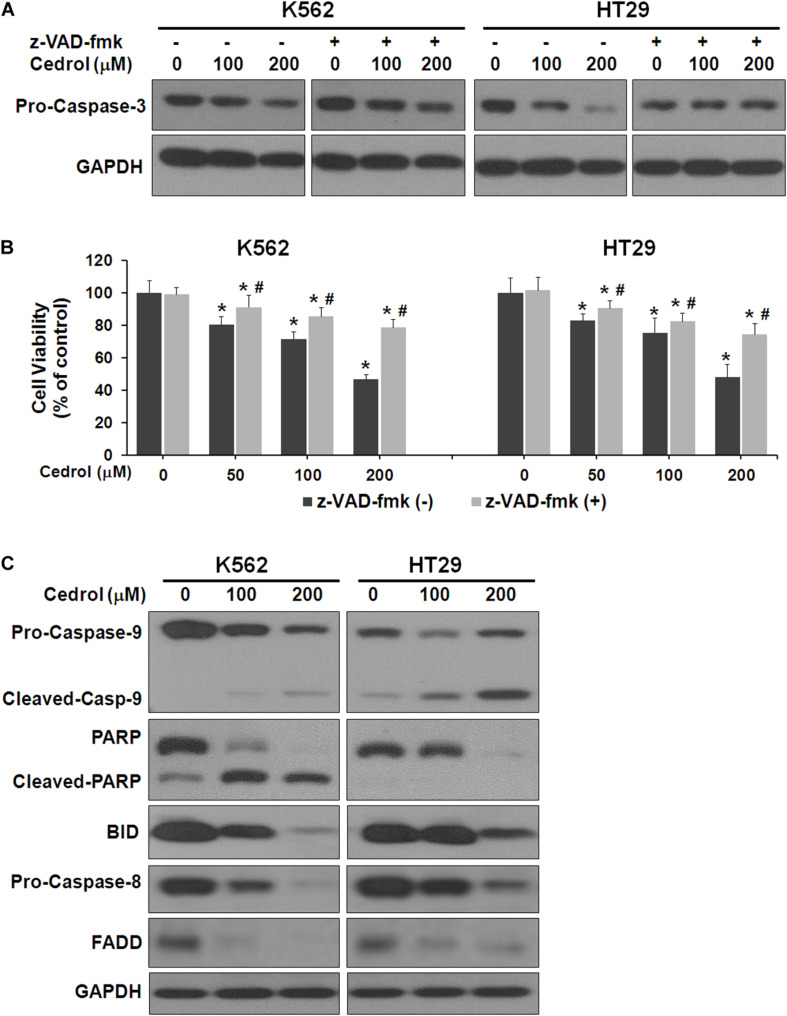
Cedrol induced caspase activation in K562 and HT29 cells. **(A)** Cells were treated with z-VAD-fmk (50 μM) for 1 h in serum-free media. Cells were then washed with either VH or cedrol in serum-free media for 18 h. Protein was isolated, and Western blotting was performed. **(B)** Cells were treated with or without cedrol in combination of z-VAD-fmk (50 μM) for 48 h. Cell viability was assayed as described in Materials and Methods. **(C)** K562 and HT29 cells were treated with VH or cedrol for 18 h. Total cytosolic proteins were isolated, and Western blotting was performed.

Next, we performed experiments to study the mechanisms by which cedrol induced cell death. Firstly, we examined the effect of cedrol on proteins in apoptosis cascade in the presence and absence of z-VAD-fmk (pan-caspase inhibitor). Treatment of K562 and HT29 cells by cedrol showed activation of caspase-3 in a dose-dependent manner. Addition of z-VAD-fmk to cells inhibited the caspase-3-activating effect of cedrol (Figure 2A). We also assessed the viability of cells treated with cedrol in combination with z-VAD-fmk. Addition of z-VAD-fmk restored the cedrol-mediated suppression of cell viability in K562 and HT29 cells but not completely (Figure 2B). The reason behind this observation could be partial necrosis induced by cedrol especially at 200 μM, as shown in Figure 1B. These results suggest that cedrol suppresses growth of cancer cells through induction of apoptosis as a mechanism, but other growth regulatory pathways may also be associated. Secondly, we determined the apoptosis pathway (intrinsic or extrinsic) activated by cedrol. As seen in Figure 2C, the level of BID (BH3-interacting-domain death agonist) was dramatically suppressed by cedrol. BID is a pro-apoptotic member of the Bcl-2 protein family which is known to initiate the mitochondrial intrinsic pathway of apoptosis. Cedrol further caused the activation of caspase-9 and subsequently caspase-3 in a dose-dependent manner. Caspase-3 then executes cell death by cleavage of key nuclear substrate PARP (poly ADP ribose polymerase) (Figure 2C). These results confirm that cedrol induced mitochondrial intrinsic apoptosis in K562 and HT29 cells. Studies using sesquiterpenes like resveratrol, costunolide, and tehranolide have shown anticancer activities through the Fas/CD95-caspase-8-dependent extrinsic pathway of apoptosis (Reis-Sobreiro et al., 2009; Choi et al., 2012; Noori and Hassan, 2012). Resveratrol induced a co-clustering of the raft protein contents and death-induced silencing complex (DISC) constituents: Fas/CD95, FADD (Fas-associated protein with death domain), and procaspase-8 (Reis-Sobreiro et al., 2009). Unlike other sesquiterpenes, cedrol inhibited the levels of FADD in K562 and HT29 cells, which rules out its possibility to be recruited in the DISC. However, pro-caspase-8 was inhibited by cedrol especially at a higher concentration (200 μM) which may be a non-specific activation, which often occurs in the intrinsic apoptosis pathway.

### Cedrol Suppressed AKT/ERK/mTOR and NF-κB Signaling Pathways in K562 and HT29 Cells

**FIGURE 3:**
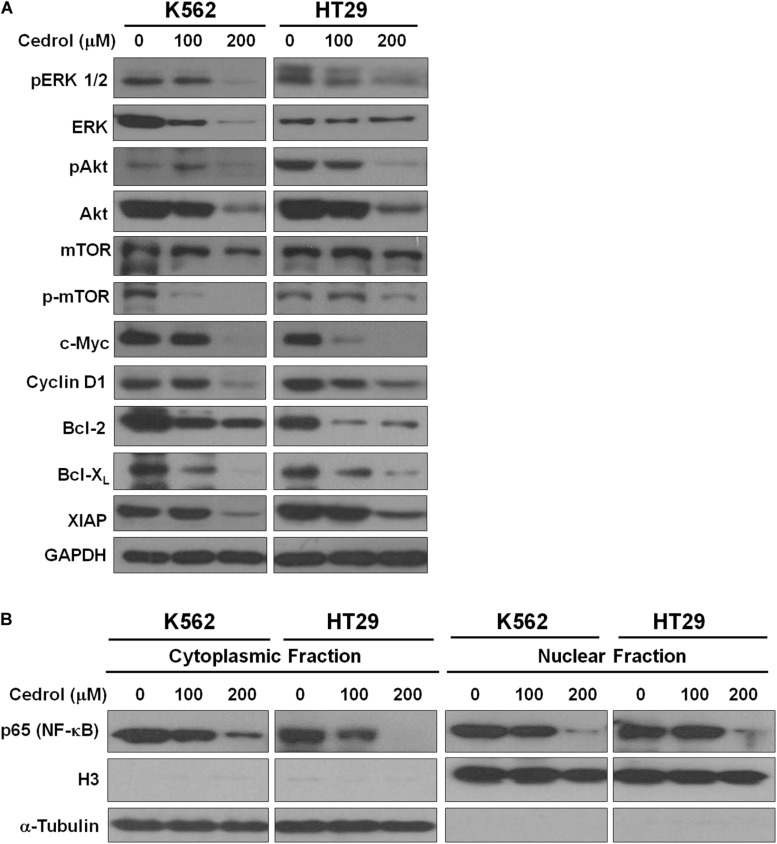
Cedrol inhibited the levels of proteins in pro-survival signaling pathways. **(A)** K562 and HT29 cells were treated with either VH or cedrol for 18 h. Proteins were isolated, and Western blotting for proteins related to cell survival signaling and apoptosis was performed. **(B)** K562 and HT29 cells were treated with either VH or cedrol for 18 h. Cytosolic and nuclear protein lysates were isolated and Western blotting for the NF-κB-p65 subunit was performed. Histone protein H3 and GAPDH were detected as a loading control in nuclear and cytoplasmic lysates, respectively.

Although cedrol treatment could sensitize K562 and HT29 cells to apoptosis and partial necrosis, the pan-caspase inhibitor (z-VAD-fmk) could not fully abrogate the effects of cedrol. Therefore, we attempted to search other mechanistic possibilities associated with growth inhibitory effects of cedrol in K562 and HT29 cell lines. Analysis of proteins in cell survival signaling pathways shows that cedrol inhibited the levels of pERK 1/2 and pAkt and pmTOR proteins in a dose-dependent manner (Figure 3A). Cedrol further inhibited the levels of anti-apoptosis proteins of the Bcl-2 family (Bcl-2 and Bcl-X_*L*_) as well as XIAP. It is also noteworthy that cedrol remarkably inhibited the levels of cyclin D1 and c-Myc in both cell lines (Figure 3A). The expression of Bcl-2 family proteins is under the transcriptional control of both the ERK 1/2 and NF-κB signaling pathways. Thus, we examined the levels of NF-κB protein in K562 and HT29 cells treated with cedrol. We found that cedrol inhibited the levels of the p65 subunit of NF-κB protein in cytoplasmic as well as nuclear lysates in a dose-dependent manner (Figure 3B). The effect of cedrol on downregulation of NF-κB was also confirmed by analyzing the nuclear translocation of NF-κB proteins. Cedrol effectively inhibited the nuclear levels of p65 protein as observed by a dramatic reduction at 200 μM. These findings suggest that cedrol interferes with PI3K/AKT/ERK and NF-kB signaling as additional mechanism in growth inhibition of cancer cells.

### Cedrol Causes Redistribution of Cholesterol/Sphingomyelin From Plasma Membrane Lipid Rafts

**FIGURE 4:**
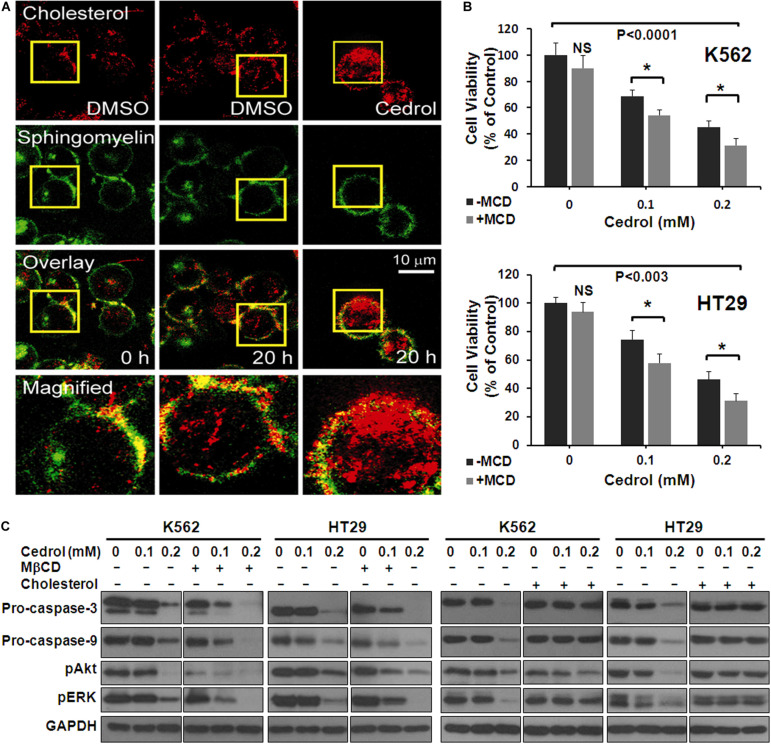
Cedrol redistributed cholesterol from membrane lipid raft. **(A)** K562 cells were treated with VH or cedrol (200 μM) for 20 h. Cells were treated with Bodipy-labeled sphingomyelin and cholesterol, then visualized under a confocal microscope. VH-treated cells were visualized at 0 h to imagine the basal membrane organization. Yellow boxes represent the area magnified. **(B)** K562 and HT29 cells were treated with 2.5 mg/ml methyl-β-cyclodextrin (MβCD) for 2 h in serum-free medium. Cells were then treated with either VH or cedrol in serum-free media for 48 h, and cell viability was assayed. **(C)** K562 and HT29 cells were treated with or without MβCD (2.5 mg/ml), cholesterol (10 μg/ml), and cedrol for 18 h. Protein isolation and Western blotting were performed.

In order to explore the mechanism underlying the effects of cedrol, we assessed the effect on the organization of cell membranes. K562 cells were treated with Bodipy-labeled sphingomyelin and cholesterol followed by confocal fluorescence microscopy imaging (Figure 4A). The control-treated cells displayed a preferential localization of both cholesterol and sphingomyelin in certain areas of the plasma membrane. Cedrol treatment (200 μM) caused a redistribution of both lipid contents across the membrane surface. These observations suggest that cedrol alters the distribution of cholesterol and sphingomyelin contents in the plasma membrane. Since cholesterol and sphingomyelin are key components in the membrane raft domains (Edidin, 2001; van Deurs et al., 2003), these alterations by cedrol could be associated with the integrity of lipid raft domains of the membrane. To test this possibility, we examined the effect of cedrol in combination with a known lipid raft-disrupting agent MβCD (2.5 mg/ml). Addition of MβCD caused about 14% more cell death in K562 cells as compared to cedrol alone (Figure 4B). In HT29 cells, MβCD caused 16.6% and 15% more cell death as compared to cedrol alone at 100 and 200 μM, respectively (Figure 4C). These results showed the additive combination effects of cedrol and MβCD on growth inhibition. We also examined the levels of proteins in apoptosis cascade and AKT/ERK signaling when treated in combination with cedrol and MβCD. The combination of cedrol and MβCD showed a more exaggerated response as compared to cedrol alone. Apoptosis-related caspases (9 and 3) and cell survival-related proteins (pAkt and pErk) were highly inhibited in combination as compared to cedrol alone (Figure 4C). In addition to the lipid raft disruption approach, we also utilized the cholesterol replenishing approach using the cholesterol:MβCD complex prepared by the previously described method (Christian et al., 1997). Our results show that treatment of cells with the cholesterol complex (10 μg/ml) dramatically attenuated the effects of cedrol (Figure 4C). K562 and HT29 cells treated with the cholesterol complex showed lesser inhibition of pro-caspases (9 and 3) and pAkt and pErk as compared to cedrol alone (Figure 4C). It is thus evident that lipid raft disruption exaggerates, and cholesterol replenishment abrogates, the effects of cedrol. These results indicate two clear possibilities that effects of cedrol are highly dependent on the integrity of membrane lipid raft and that apoptosis induction and suppression of pro-survival signaling act as parallel mechanisms.

### Cedrol Suppressed the ROS Level Through Inhibition of Nox2 Activity

**FIGURE 5:**
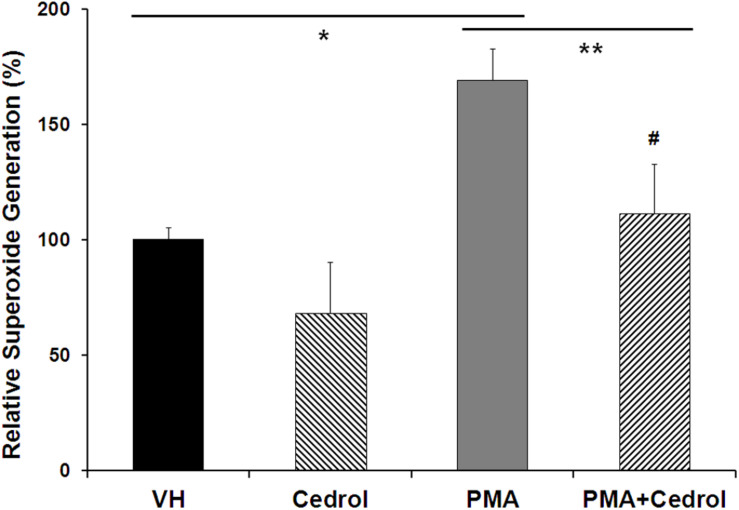
Cedrol suppressed intracellular ROS generation in K562 cells. K562 cells were treated with either VH or cedrol (200 μM) for 18 h and then exposed to 0.3 μM phorbol 12-myristate 13-acetate (PMA) for 5 min. DCFH-DA (10 mM) assay was performed, and the fluorescence of product DCFHA was measured by flow cytometry. Values expressed are means ± SD of percent relative fluorescence units for three independent experiments. *P vs. VH; **P vs. VH + PMA; #non-significant vs. VH (vehicle control).

Nox2 is a membrane-bound member of the NADPH oxidase enzyme complex, an important source of non-mitochondrial intracellular ROS generation (Leto et al., 2009). We utilized DCFH-DA assay in combination with phorbol 12-myristate 13-acetate (PMA), specific for detection of Nox2 activity-dependent ROS generation (Figure 5). PMA, a diester of phorbol, functions as a tumor promoter via activation of PKC signaling. PMA is a known and specific activator of Nox2, which stimulates cells for ROS generation through phosphorylation of p47phox sites of Nox2 (Leto et al., 2009; Jeon et al., 2010). Recently, it has been demonstrated that the potential cytosolic tail of Nox2 was phosphorylated during PMA activation by a PKC-dependent mechanism (Raad et al., 2009). Treatment of K562 cells with cedrol (200 μM) significantly inhibited the generation of ROS to 68% (*P* = 0.033) as compared to vehicle control (100%). PMA-treated K562 cells showed high fluorescence intensity (169%, *P* = 0.0004), indicating significantly enhanced production of ROS. Treatment of PMA-sensitized cells with cedrol inhibited the relative superoxide generation to near the level of control (111%). This inhibition of ROS generation was highly significant as compared to PMA-treated cells (*P* = 0.0081) but not significant to vehicle control (*P* = 0.20). Cedrol showed a reduction in ROS even without treatment with Nox2 activator PMA, yet with lesser statistical significance (*P* = 0.033). However, in K562 cells treated with Nox2 activator PMA, the reduction in ROS was statistically highly significant (*P* = 0.0081). This assay demonstrates that inhibition of ROS generation by cedrol is possibly mediated by the inhibition of Nox2 activity. Since Nox enzyme complexes are located in the lipid raft-rich region of the plasma membrane (Leto et al., 2009), the effect of cedrol appears to be dependent on the lipid raft-destabilizing property. Considering that high levels of ROS are functional features of cancer cell growth promotion, these properties of cedrol are likely to be associated with growth inhibition of cancer cells.

### Cedrol Induced Ceramide Synthesis in K562 Cells

**FIGURE 6:**
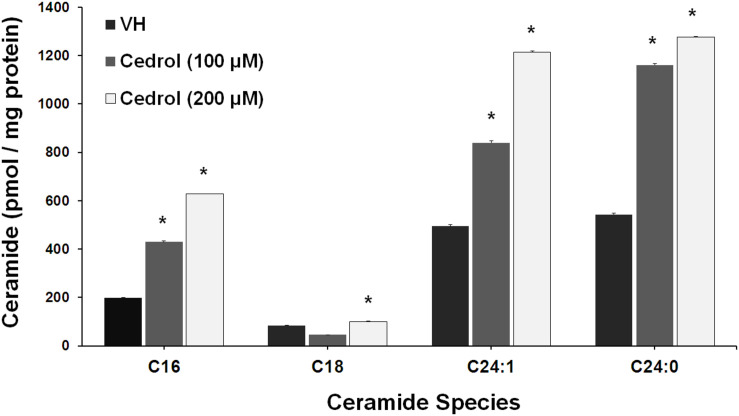
Cedrol enhanced ceramide production in K562 cells. K562 cells treated with VH and cedrol for 18 h. Protein was isolated, and a total of 200 μg protein lysates was used for ceramide species quantification. Levels of C16, C18, C24, C24:1, and C24:0 ceramide species were determined by LC-MS/MS. Data are mean ± SD of three independent experiments. *P vs. VH (vehicle control).

Ceramide production has been found in disrupted plasma membrane and then exerted a pro-apoptotic response in cancer cells (Ogretmen and Hannun, 2004). We further analyze the lipid raft-dependent pro-apoptotic mechanism of cedrol by quantifying the intracellular ceramide species in K562 cells by LC-MS/MS (Figure 6). Incubation of K562 cells with cedrol for 18 h led to a dose-dependent increase in intracellular accumulation of ceramide species (C16, C18, C24:1, and C24:0). Ceramide can be produced via multiple mechanisms including through the hydrolysis of sphingomyelin by acid and neutral sphingomyelinase or by a *de novo* synthesis through a pathway involving the ceramide synthase (Ogretmen and Hannun, 2004). Ceramide was reported to act as tumor-suppressor lipid through activation of apoptosis and suppression of cell survival (Pettus et al., 2002; Huang et al., 2011), especially limiting the PI3K signaling (Kitatani et al., 2016). Ceramide was also shown to affect the stress-induced ceramide on apoptosis through PI3K downregulation. Ceramide induction directly downregulated the levels of PI3K, which resulted in inhibition of Akt phosphorylation (Zundel and Giaccia, 1998). This suggested that ceramide levels could act as a general apoptotic rheostat and control cell survival through PI3K and Akt. This study showed similar observations with the treatment of cancer cells with cedrol, which caused the induction of the ceramide product and resulted in apoptosis activation and suppression of cell survival. These observations indicate that ceramide biosynthesis acts as a plausible mechanism for growth inhibition and that it was apparently dependent on membrane lipid raft destabilization.

## Discussion

Growing evidence suggests that the combination of phytochemicals with synthetic agents may serve as an effective anticancer therapeutic strategy including stress or drug-induced apoptotic signaling in tumor cells mediated through lipid rafts (Gajate et al., 2009; Leto et al., 2009; Bae et al., 2011; Ali et al., 2020). Reportedly, cancer cell lipid composition differs from the non-malignant cell profile but is variable between malignancy types (Bernardes and Fialho, 2018; Casares et al., 2019; Pakiet et al., 2019). Also, the molecular dynamics of the plasma membrane in cancer cells showed the loss of lipid asymmetry leading to a decreased permeability and resistance in response to cisplatin chemotherapy (Rivel et al., 2019). Thus, the disorganization of lipid domains in the membrane can significantly alter cell signaling pathways in glioma cancer (Rivel et al., 2019). This study showed that cedrol suppresses the growth of human leukemia (K562) and colon cancer (HT29) cell by a central mechanism of membrane lipid raft destabilization which increased the biosynthesis of ceramide and led to induction of apoptosis. The ceramide-dependent pathway is a pro-growth, but it becomes pro-apoptosis in the presence of dephosphorylated Bad and cleaved BID (Megha and London, 2004). Similarly, cedrol was found to induce mitochondrial intrinsic apoptosis by activating BID cleavage followed by activation of caspases-9 and -3.

Studies using sesquiterpenes like resveratrol, artemisinin, thapsigargin, and parthenolide have shown apoptosis induction through the extrinsic pathway by recruiting FasL/CD95-FADD (Reis-Sobreiro et al., 2009; Noori and Hassan, 2012; Islam et al., 2018). Disruption of lipid rafts by MβCD has been shown to inhibit the resveratrol-induced formation of Fas/CD95 clusters and apoptosis (Reis-Sobreiro et al., 2009). Edelfosine, an anticancer drug, has also shown the involvement of DISC clusters in lipid raft aggregates for the induction of apoptosis in leukemic cells (Gajate et al., 2009).

Lipid rafts are known to transduce several cell growth signals and other physiological processes including ROS generation (Megha and London, 2004; Malorni et al., 2008). NADPH oxidase enzymes (Nox1, Nox2, and Nox4), located in lipid rafts, have been found associated with growth promotion in cancer (Leto et al., 2009; Rao Malla et al., 2010; Bae et al., 2011). Cedrol was found to suppress the generation of ROS in K562 cells to some extent and more significantly in PMA-sensitized cells (Figure 5). Cedrol suppresses ROS generation possibly through inhibition of Nox2 activity, which may be resultant of lipid raft disruption because Nox activation requires a protein–protein interaction between cytoplasmic components and raft-associated subunits (Shao et al., 2003). Growth inhibition of breast cancer cells was found to be mediated by downregulation of Nox2-associated lowering of ROS through lipid raft disruption (Leto et al., 2009; Rao Malla et al., 2010). The integrity of lipid rafts thus appears to play a key role in regulating Nox2-mediated ROS generation and growth promotion in cancer cells. ROS favors the growth of cancer cells via upregulation of the pathways like ERK and NF-κB (McCubrey et al., 2007; Trachootham et al., 2008). NF-κB is a major transcription factor critical in controlling cell proliferation and apoptosis (Wu and Zhou, 2009). Reportedly, sesquiterpenes are a potential source for NF-κB inhibition (Ghantous et al., 2010). Cedrol also inhibited the phosphorylation of the p65 subunit of NF-κB at both cytoplasmic and nuclear levels (Figure 4B). Cedrol downregulated the expression of NF-κB at the cytosolic level and suppressed its nuclear translocation plausibly due to reduction in the cytosolic NF-κB-p65 and inhibition of its nuclear translocation.

PI3K/Akt pathways promote cell survival by directly or indirectly inhibiting apoptosis through phosphorylation/inactivation of Bad and caspase-9 and other anti-apoptosis proteins like XIAP (Datta et al., 1997; Breckenridge and Xue, 2004). Ceramide is also known to activate protein phosphatases which can inactivate survival signaling such as PI3K/AKT/mTOR (Ruvolo, 2003). Cedrol suppressed the levels of pAkt followed by the activation of BID and inhibition of XIAP (Figures 2C, 3A). One of the major effectors downstream of Akt is glycogen synthase kinase-3 (GSK3) which regulates cell cycle progression by phosphorylating cyclin D1 (Diehl et al., 1998). Cedrol subsequently inhibited the levels of cyclin D1 and c-Myc (Figure 3A). Reduction in pAkt and pErk by cedrol was confirmed to be dependent on lipid raft alterations by using MβCD and cholesterol (Figures 4B,C). Cedrol was shown to inhibit proliferation of lung cancer A549 cells through suppression of PI3K/Akt signaling pathways and induction of autophagy via increase in intracellular ROS production (Zhang et al., 2016). Simvastatin (a cholesterol synthesis inhibitor) has been shown to induce apoptosis in prostate cancer cells by lowering the raft cholesterol content and inhibiting Akt/PKB pathway signaling. Replenishing cell membranes with cholesterol reversed the inhibitory and apoptotic effects of simvastatin (Zhuang et al., 2005). Cholesterol homeostasis was also found to be associated with the activity of mTOR signaling. Itraconazole (inhibitor of angiogenesis) led to the inhibition of mTOR activity in endothelial cells, which was partially restored by extracellular cholesterol delivery (Xu et al., 2010). Euphol, a euphane-type triterpene alcohol (ıC_30_H_50_O), obtained from *Euphorbia tirucalli* was found to negatively regulate the expression of TGF-β responsiveness via TGF-β receptor segregation inside membrane rafts (Chen et al., 2015). Euphol is structurally similar to cholesterol and has shown a wide range of pharmacological properties especially anti-inflammatory and anticancer effects in basal cell carcinomas, leukemia, and lung, prostate, and breast cancers. Recently, 10-gingerol (C_21_H_34_O_4_), a β-hydroxy ketone and a phenolic member of monomethoxybenzene, was found to modulate the lipid rafts of breast cancer MDA-MB-231/IR cells. It caused the redistribution of cholesterol content of the lipid rafts and rafts and attenuated the key PI3K/Akt signaling components in lipid rafts of cells (Ediriweera et al., 2020). Another gingerol derivative (6-gingerol) was shown to modulate a variety of classical signaling cascades in different types of cancers especially ERK, JNK, PI3-K/Akt, PLC-γ1, and JAK/STAT pathways (de Lima et al., 2018). The PI3K/Akt signaling modulation was associated with lipid raft-associated modification induced by 10-gingerol which was similarly observed in this study using cedrol.

**FIGURE 7:**
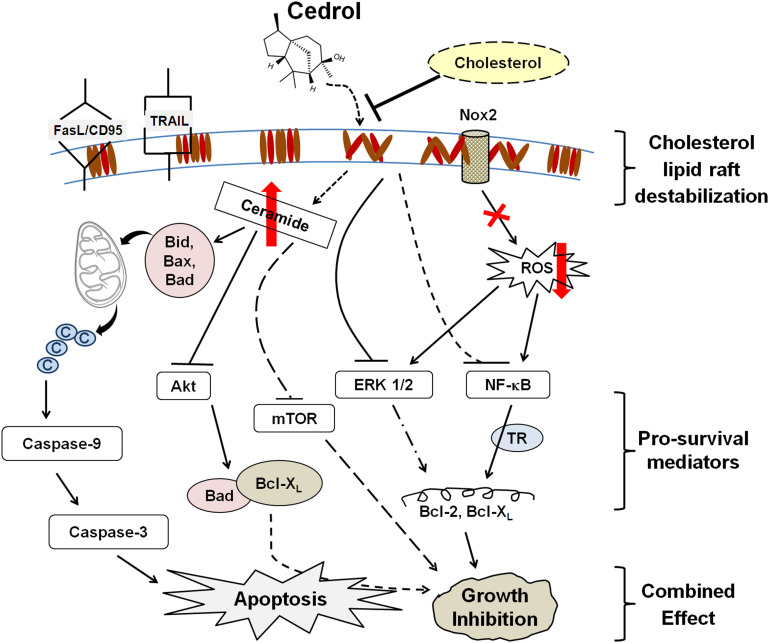
Schematic sumamry of the molecular signaling triggered by cedrol. Cedrol depletes cholesterol from membrane lipid raft and leads to modulation of various cellular biochemical signaling events within cell. ©, cytochrome c; TR, transcriptional regulation.

Our observations (as summarized in Figure 7) collectively suggest that cedrol-mediated growth inhibition is a combined effect of inhibition of pro-survival signaling and induction of apoptosis due to lipid raft disruption which is supported by other existing reports. This study first demonstrates that cedrol may act as a natural lipid raft-destabilizing agent with potentials to inhibit the growth of human cancer cells. Cedrol caused the redistribution of cholesterol from the membrane lipid raft which initiated two major biochemical events: production of ceramide and inhibition of Nox2-dependent ROS generation. Ceramide biosynthesis and inhibition of ROS generation initiated the cascade of molecular events leading to the induction of apoptosis. Ceramide is known to act as a tumor-suppressor lipid through its interaction with a variety of stress stimuli leading to apoptosis (Huang et al., 2011). Increase in intracellular ceramide is associated with initiation of intrinsic apoptotic signaling, and defects in ceramide generation and sphingolipid metabolism are further correlated with cancer cell survival and cancer therapy resistance (Pettus et al., 2002). Ceramide was identified as a bioactive lipid that limits PI3K-controlled cell motility and as metastasis-suppressor in ovarian cancer (Kitatani et al., 2016) which indicates the plausible translational measure to develop ceramide-based therapy for metastatic diseases. The main bioactive sphingolipids like ceramide, sphingosine, and sphingosine 1-phosphate (S1P) are associated with key biological functions in regulation of cellular homeostasis. A variety of alkaline ceramidases (ACER1-3) were associated with Farber’s disease and involved in the regulation of cell viability in the small intestine and colon cancer (Coant et al., 2017), yet these activities were not recorded in treatment with cedrol. Our findings demonstrate that cedrol suppressed pro-survival signaling by inhibiting proteins in PI3AKT/mTOR/ERK1/2 and NF-κB signaling pathways through lipid raft destabilization. This study provides significant evidence for the biological basis of using cedrol as a supplement for cancer prevention through lipid raft destabilization.

## Materials and Methods

### Cell Cultures and Treatment

K562 and HT29 cell lines purchased from the American Type Culture Collection (ATCC) were maintained in RPMI 1640 medium (Gibco Invitrogen, Grand Island, NY, United States) supplemented with 10% fetal bovine serum (Hyclone, Logan, UT, United States), 2 mM L-glutamine, 100 units/ml penicillin, and 100 μg/ml streptomycin (Gibco Invitrogen) at 37°C in humidified atmosphere of 5% CO_2_. Cedrol was purchased from Sigma Chemicals.

### Cell Viability Assays

K562 and HT29 cells (4000 cell/well) were seeded in 96-well plates and treated with cedrol for 48 h. Cell viability in K562 cells was assessed by using Ez-Cytox WST assay kit (Daeil Lab, Seoul, South Korea) according to the manufacturer’s instructions. MTT [3-(4,5-dimethylthiazol-2-yl)-2,5-diphenyltetrazolium bromide] assay (Biosesang Inc., South Korea) was performed in HT29 cells, as previously described (Mishra et al., 2013a, b). Optical densities of WST and MTT assay products were measured at 460 and 570 nm using a microplate reader (Epoch Micro-Volume Spectrophotometer System, United States).

### Flow Cytometric Analysis of Apoptosis

Apoptosis induction was analyzed by annexin V/7-amino-actinomycin (7-AAD) double staining using Annexin V-FITC Apoptosis Detection Kit II (BD Bioscience, San Jose, CA, United States) according to the manufacturer’s instructions and as described earlier (Mishra et al., 2013a, b). K562 cells (2 × 10^6^/ml) were treated with cedrol for 18 h followed by annexin V/7-AAD staining as per standard protocol and then flow cytometry analysis of apoptosis using FACSCalibur (BD Bioscience, San Jose, CA, United States).

### DNA Fragmentation Assay for Apoptosis

DNA fragmentation was assessed by electrophoresis of genomic DNA extracted from K562 cells as described previously (Muller et al., 1996). Briefly, K562 cells (2 × 10^6^/ml) treated with cedrol for 18 h were harvested and homogenized with 50 μl of lysis buffer (50 mM Tris–HCl, 1% NP-40, 20 mM EDTA). The supernatant collected from whole-cell lysate was treated with 5 μg RNase A and 1% SDS for RNA digestion at 56°C for 2 h, subsequently with 2.5 μg/μl proteinase K for protein digestion at 37°C for 2 h. DNA was precipitated using 10 M ammonium acetate and ethanol, dissolved in gel loading buffer, and electrophoresed on 1% agarose gel with ethidium bromide staining.

### Western Blotting for Protein Analysis

K562 and HT29 cells (2 × 10^5^/ml) were treated with cedrol for 18 h. Preparation of whole-cell lysates, protein quantification, gel electrophoresis, and Western blotting were performed as previously described (Mishra et al., 2013a, b). Nuclear protein lysates were prepared by homogenizing cells in lysis buffer mixed with Igepal CA-360 solution (0.6%). The cell homogenate was centrifuged, and the cytoplasmic fraction was collected as supernatant. The cell pellet was further treated with extraction buffer and centrifuged to collect the supernatant containing the nuclear fraction. Equivalent amounts of protein from cell and nuclear lysates were resolved by SDS-PAGE and immunoblotted with primary antibodies. GAPDH was detected as loading control. Bands were detected using ECL Western blotting detection reagents (AbFrontier, Seoul, South Korea).

### Confocal Imaging of Cholesterol–Lipid Raft in Cells

Fluorescent Bodipy-labeled analogs of sphingolipids and cholesterol are specifically utilized to study the lipid microdomains of the cell membrane. Bodipy-labeled sphingomyelin and cholesterol can visualize the membrane lipid microdomains in living cells based on their fluorescence properties and help in examining the relationship between membrane domains at the cell surface (Marks et al., 2008). K562 cells were treated with vehicle control or cedrol for 20 h and collected by centrifugation at 2000 rpm for 3 min at 4°C. The pellet was resuspended in serum-free medium and then incubated with 20 μM of each Bodipy-labeled sphingomyelin and cholesterol (Invitrogen, United States) in serum-free medium for 30 min at 4°C. Cells were then washed with cold serum-free medium and observed under a confocal microscope.

### Superoxide Generation Assay

Non-mitochondrial production of ROS was analyzed to assess the effect of cedrol on the plasma membrane-dependent enzyme activity of Nox2. K562 cells (2 × 10^6^/ml) were treated with cedrol for 18 h and stimulated with 0.3 μM phorbol-12-myristate 13-acetate (PMA) to induce generation of ROS. Five minutes later, cells were harvested and incubated with 10 mM of redox-sensitive dye 2’,7’-dichlorodihydrofluorescein diacetate (DCFH-DA) (Molecular Probes, Invitrogen, United States) for 30 min in the dark at 37°C. DCFHA fluorescence was measured by flow cytometry using FACSCalibur (BD Bioscience, San Jose, CA, United States).

### LC-MS/MS Analysis for Ceramide Production

Ceramide quantification was performed by LC-MS/MS technique on a Shimadzu Prominence UPLC system (Shimadzu, Kyoto, Japan) equipped with a thermostatted autosampler, binary pump, degasser, and thermostatted column compartment. The chromatographic separation was performed on an XTerra MS C18 column (2.1 × 50 mm, 5 μm particles; Waters Corporation, Milford, MA, United States) with a SecurityGuard C18 guard column (4 mm × 20 mm I.D.; Phenomenex, United States). MS analysis was performed on an Applied Biosystems API2000 triple-quadrupole mass spectrometer by multiple reaction monitoring (MRM) in positive and negative electrospray ionization mode. Quantification of each compound was achieved by comparison of the analyte/internal standard peak area ratios using C16, C18, C20, C24:1, and C24:0 ceramide mixtures (0–2000 pmol) as calibration standards. Acquisition and analysis of data were performed with Analyst^TM^ software (version 1.4.2, Applied Biosystems, Foster City, CA, United States). K562 cells treated with either vehicle control or cedrol were harvested and total 200 μg protein cell lysates were used for ceramide species quantification.

### Statistical Analyses

All values are expressed as mean with standard deviations (SD). Data were analyzed using SPSS v17 and Systat SigmaPlot 10.0 statistical software. Statistical significance was calculated by Student’s *t*-test and one-way analysis of variance (ANOVA) for multiple comparisons. *P* < 0.05 was considered statistically significant.

## Data Availability Statement

The original contributions presented in the study are included in the article/Supplementary Material, further inquiries can be directed to the corresponding author/s.

## Author Contributions

HK and SO performed conception and design of studies. SM, YB, YL, and JK performed acquisition, analysis, and interpretation. SM, HK, and SO performed critical review, discussion, and manuscript preparation. All authors contributed to the article and approved the submitted version.

## Conflict of Interest

The authors declare that the research was conducted in the absence of any commercial or financial relationships that could be construed as a potential conflict of interest.

## Supplementary Material

Supplementary Figure 1 Effect of cedrol on cell cycle progression in K562 cells. K562 cells were treated with indicated concentrations of VH or cedrol for 18 h. Cells were treated with propidium iodide (PI) and then analyzed by flow cytometry. Percentage of cells in G1/G0, S, and G2/M phases were determined. Values represented on top of each box represent percentage of cells in difference phases of cell cycle (subG1: G1: S: G2/M).
