# Supplementary figures and images for: Sesquiterpene Alcohol Cedrol Chemosensitizes Human Cancer Cells and Suppresses Cell Proliferation by Destabilizing Plasma Membrane Lipid Rafts
Source: Front Cell Dev Biol. 2021 Jan 21;8:571676. doi: 10.3389/fcell.2020.571676 (PMC7874189; doi:10.3389/fcell.2020.571676)

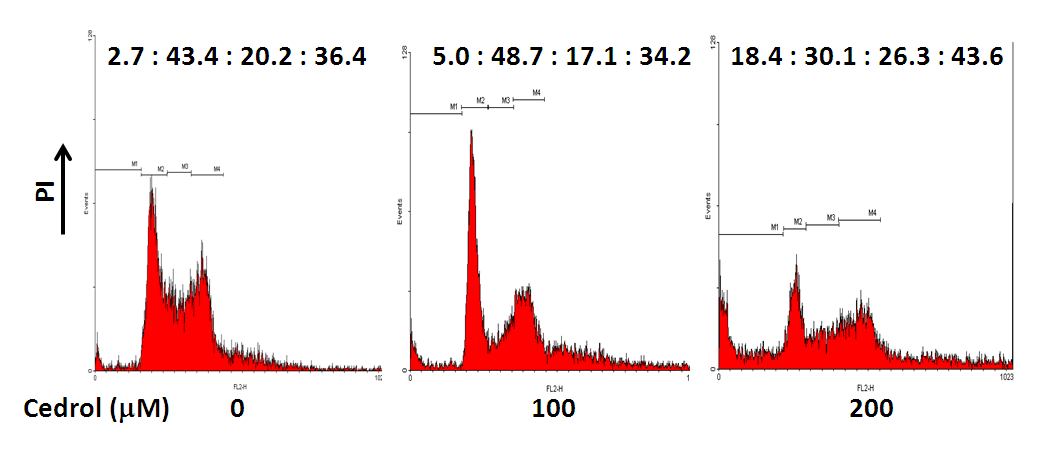

Supplement: Supplementary Figure 1 — Effect of cedrol on cell cycle progression in K562 cells. K562 cells were treated with indicated concentrations of VH or cedrol for 18 h. Cells were treated with propidium iodide (PI) and then analyzed by flow cytometry. Percentage of cells in G1/G0, S, and G2/M phases were determined. Values represented on top of each box represent percentage of cells in difference phases of cell cycle (subG1: G1: S: G2/M). [file Image_1.TIF]
