# Supplementary material for: Sesquiterpene Alcohol Cedrol Chemosensitizes Human Cancer Cells and Suppresses Cell Proliferation by Destabilizing Plasma Membrane Lipid Rafts
Source: Front Cell Dev Biol. 2021 Jan 21;8:571676. doi: 10.3389/fcell.2020.571676 (PMC7874189; doi:10.3389/fcell.2020.571676)
Supplement: Supplementary file 2 [file Table_1.DOC]

**SUPPLEMENTARY TABLE 1** Effect of cedrol on growth of human cancer cell lines of different lineage.

| Cell Line | Cell Proliferation (% of control) at cedrol | | | | GI50* |
| --- | --- | --- | --- | --- | --- |
|  | 50 μM | 100 μM | 200 μM | 400 μM |  |
| K562 | 79 | 69 | 47 | 34 | 179.5 |
| HT29 | 85 | 74 | 46 | 30 | 185.5 |
| NUGC-3 | 100 | 85 | 53 | 16 | 208.2 |
| MDA-MB-231 | 95 | 82 | 58 | 16 | 214.7 |
| MKN28 | 96 | 78 | 72 | 71 | >400 |
| HCT15 | 96 | 94 | 83 | 93 | >400 |
| HCT116 | 102 | 95 | 91 | 87 | >400 |
| A549 | 105 | 96 | 88 | 91 | >400 |
| NCI-H23 | 100 | 92 | 68 | 66 | >400 |
| DU145 | 91 | 89 | 68 | 74 | >400 |
| PC-3 | 94 | 89 | 77 | 74 | >400 |
| SK-OV-3 | 99 | 77 | 94 | 93 | >400 |
| Caki-1 | 97 | 94 | 82 | 79 | >400 |

* GI50 was calculated as the concentration (μM) of cedrol that achieved 50% growth inhibition of cells.
